# Einfluss einer adjuvanten Strahlentherapie im Vergleich zur „Early-salvage-Strahlentherapie“ auf die Letalität von Männern mit einem hohen Rezidivrisiko nach radikaler Prostatektomie wegen Prostatakarzinom

**DOI:** 10.1007/s00066-021-01882-2

**Published:** 2021-11-25

**Authors:** Simon K. B. Spohn, Anca-Ligia Grosu

**Affiliations:** Klinik für Strahlenheilkunde, Universitätsklinikum Freiburg, Robert-Koch-Str. 3, 79106 Freiburg, Deutschland

## Hintergrund

2020 wurden die Ergebnisse von drei kontrollierten, randomisierten Phase-III-Studien (RCT) veröffentlicht, die eine adjuvante Strahlentherapie (aRT) mit einer Early-salvage-Strahlentherapie (sRT) nach Operation eines Prostatakarzinoms (PCa) verglichen. Die GETUG-AFU-17- [1] und die RADICALS-RT-Studie [2] untersuchten die Überlegenheit einer aRT mittels der primären Endpunkte progressionsfreies Überleben (PFS) und metastasenfreies Überleben (MFS), während die RAVES-Studie [3] die Nichtunterlegenheit der sRT mittels des Endpunkts PFS untersuchte. Diese Studien konnten keinen signifikanten Unterschied des PFS zwischen aRT und sRT identifizieren. Mit einer Hazard Ratio (HR) von > 1 in RADICALS-RT und in Hochrisikosubgruppen von Patienten mit pT3b-Tumorstadien und Gleason-Score ≥ 8 in RAVES legen die Ergebnisse sogar nahe, dass eine sRT einer aRT hinsichtlich des PFS überlegen ist.

Eine mögliche Erklärung für diese Ergebnisse ist der „immortaltime bias“, der entstehen kann, wenn in einem Zeitraum das zu beobachtende Ereignis einer Kohorte gar nicht auftreten kann. Im Fall der RCT begann die 2 Monate dauernde Behandlung von Patienten im aRT-Arm bereits bei undetektierbarem PSA-Wert, während die Behandlung von Patienten im sRT-Arm innerhalb von 4 Monaten nach Überschreitung des jeweiligen PSA-Schwellenwerts (> 0,1 in der RADICALS-RT-Studie und > 0,2 ng/ml in den anderen Studien) initiiert wurde und die Patienten hinsichtlich des Progresses innerhalb von 3 Monaten nach durchgeführter sRT analysiert wurden. Folglich kann ein Krankheitsprogress von Männern im sRT-Arm innerhalb einiger Monate nach Überschreiten des PSA-Schwellenwerts nicht detektiert werden. Unter der Annahme, dass Patienten mit ungünstigen pathologischen Merkmalen (pN1, Gleason-Score 8–10, ≥ pT3a) im Falle eines Rezidivs einen rasch ansteigenden PSA-Wert mit einer Verdopplungszeit von < 7,5 Monaten aufweisen können, ist das Erreichen eines PSA-Werts von 0,4 ng/ml, was bereits einem Progress entsprechen würde, innerhalb des Zeitraums möglich, in dem die sRT geplant und/oder durchgeführt wird. Somit würde der Progress im sRT-Arm erst zu einem späteren Zeitpunkt diagnostiziert werden als im aRT-Arm. Das erklärt möglicherweise, warum eine „early sRT“ der aRT in der RADICALS-RT-Studie überlegen war.

Diesen Aspekt berücksichtigend, führten die Autoren der hier kommentierten Arbeit um Derya Tilki eine retrospektive Analyse großer multizentrischer Patientenkohorten durch, um zu beantworten, ob Patienten mit ungünstigen pathologischen Merkmalen von einer aRT gegenüber einer sRT profitieren können.

## Methoden

Die Studienkohorte beinhaltete 26.118 Patienten im medianen Alter von 62 Jahren mit einem Prostatakarzinom pT2–4 pN0 oder pN1 und M0, die zwischen 1989 und 2016 mit radikaler Prostatektomie (inkl. Lymphonodektomie) und nachfolgender aRT oder „early sRT“ in und außerhalb Deutschlands behandelt wurden (Universitätsklinik Hamburg-Eppendorf, Charité – Universitätsklinikum Berlin, Universitätsklinikum Ulm, University of California San Francisco und John Hopkins Medical Institution). Die Patienten wurden im ersten Jahr alle 3 Monate, bis zum fünften Jahr alle 6 Monate und anschließend jährlich nachgesorgt. Damit die Todesursache als prostatakarzinomspezifisch (PCSM) gewertet wurde, musste vor dem Tod ein kastrationsresistentes metastasiertes PCa auf der Basis steigender PSA-Werte und eines Testosteronlevels von < 20 ng/dl nachgewiesen werden. Der Vergleich der Verteilung klinischer Faktoren wurde mittels *Mantel-Haenszel‑χ*^*2*^*-Test* für kategoriale Kovariablen und im Fall einer kleinen Fallgröße mittels *Fisher-exact-Test* durchgeführt. Kontinuierliche Kovariablen wurden mittels *Wilcoxon-2-sample-Test* bestimmt. Mittels univariater und multivariater Cox-Regression wurde die Assoziation von aRT gegenüber „early sRT“ mit der „all-cause mortality“ (ACM) in Männern mit und ohne ungünstige Histologie untersucht. Für die Analyse wurde bei Patienten mit ungünstiger Pathologie derselbe RT-Startpunkt verwendet; allerdings wurden in dieser Analyse Patienten mit pN1 ausgeschlossen, da diese auch in den drei RCT nicht eingeschlossen worden waren. Zudem wurde der Einsatz einer Androgendeprivationstherapie (ADT) untersucht. Im Appendix beschreiben die Autoren, dass mittels eines multivariaten Interaktionsmodells von Fine und Gray der Anteil der PCSM auf die ACM untersucht wurde.

## Ergebnisse

819 Patienten (3,14 %) erhielten eine aRT innerhalb von 6 Monaten nach RP und 4601 (17,72 %) erhielten eine sRT bei einem medianen PSA von 0,3 ng/ml („interquartile range“ [IQR] 0,20–0,62). Von diesen Patienten hatten 655 eine PSA-Persistenz (PSA ≥ 0,1 ng/ml postoperativ) und wurden deshalb der sRT-Gruppe zugeordnet. Adjuvante ADT und Salvage-ADT (sADT) wurden in 352 (1,35 %) bzw. 2532 (9,69 %) angewandt. Die aRT wurde im Median 3,55 Monate nach RP durchgeführt mit einer medianen Dosis von 68,4 Gy auf Prostataloge und 45 Gy im Bereich der elektiven Lymphknoten, falls diese involviert waren (nach Maßgaben der behandelnden Ärzte). Eine adjuvante ADT erfolgte im Median 9,17 Monate lang.

1491 (5,71 %) Männer hatten einen pN1-Status, von denen 319 (21,4 %) eine aRT und 241 (16,16 %) eine adjuvante ADT erhielten. Die sADT wurde bei Progress initiiert. Patienten, inklusive pN1-Stadium, die eine aRT erhielten, hatten signifikant häufiger ≥ pT3a-Stadien (97,9 vs. 94,5 %) und positive Resektionsränder (82,7 vs. 45,7 %) als Patienten im sRT-Arm, allerdings wurde eine sADT signifikant seltener angewandt (36,2 vs. 47,5 %). Ähnliche Ergebnisse zeigten sich, wenn pN1-Patienten ausgeschlossen wurden.

Die mediane Nachbeobachtungszeit betrug 8,17 Jahre, und 539 von 2104 Todesfällen konnten dem PCa zugeschrieben werden. Patienten mit ungünstigen pathologischen Merkmalen hatten nach aRT ein signifikant niedrigeres ACM-Risiko, sowohl wenn Patienten mit pN1 ein- oder ausgeschlossen wurden (HR 0,61 bzw. 0,31, *p* = 0,01). Bei Patienten ohne ungünstige pathohistologische Merkmale war keine Signifikanz zu beobachten (*p* = ≥ 0,28). Auch nach Exklusion von Patienten mit PSA-Persistenz zeigte sich ein signifikant reduziertes ACM-Risiko. Bei Männern mit positivem Resektionsstatus im ≥ pT3a-Stadium war eine signifikante Assoziation zwischen aRT und einem reduzierten ACM-Risiko im Vergleich zur sRT zu finden (*p* = 0,0504). Die geschätzten 10-Jahres-ACM-Raten waren 13,78 %, 21,98 % und 27,32 % für Männer mit ungünstiger Pathologie inklusive pN1-PCa nach aRT, „early sRT“ und ohne RT. Bei Ausschluss von Männern mit pN1 betrugen die Werte 5,13 %, 22,15 % und 25,32 %. Männer ohne ungünstige Pathohistologie hatten geschätzte ACM-Raten von 7,82 %, 7,95 % und 8,81 %. Der Unterschied der 10-Jahres-ACM zwischen aRT und „early sRT“ für Patienten mit ungünstiger Pathologie betrug −8,20 % zugunsten der aRT (95 %-Konfidenzintervall [CI] −15,96 bis −0,43). Die im Appendix beschriebene Subanalyse zeigte, dass für die Reduktion des ACM-Risikos hauptsächlich eine Reduktion des PCMS-Risikos verantwortlich war.

## Schlussfolgerung der Autoren

Eine aRT war bei Patienten mit ungünstiger Pathohistologie inklusive pN1 oder Gleason-Score von 8 bis 10 und ≥ pT3a-Stadium im Vergleich zu einer „early sRT“ mit einer signifikanten Reduktion des ACM-Risikos assoziiert. Diese Assoziation wird durch die Tatsache verstärkt, dass Patienten, die eine aRT erhielten, ungünstigere prognostische Faktoren aufwiesen als Patienten, die eine „early sRT“ erhielten. Diese Resultate sind klinisch besonders relevant, da drei RCT und eine Metaanalyse [4] auf Basis des PFS keinen signifikanten Unterschied zwischen aRT und „early sRT“ gefunden hatten.

## Kommentar

Die Frage nach der optimalen Therapie von Patienten mit Hochrisikoprostatakarzinomen und ungünstigen histopathologischen Eigenschaften nach radikaler Prostatektomie bleibt auch nach der Veröffentlichung der Ergebnisse von drei RCT und einer Metaanalyse nicht hinreichend beantwortet. Obwohl die Studien keinen signifikanten Unterschied zwischen der Behandlung mittels aRT oder sRT zeigten, konnte nur ein kleiner Teil der eingeschlossenen Patienten tatsächlich Hochrisikosubgruppen für die Entstehung von Rezidiven zugeordnet werden. Zudem zeigen die Autoren um Derya Tilki zu Recht, dass die Möglichkeit eines „immortal time bias“ im Design der RCT bestand.

Die hier kommentierte retrospektive Studie versucht anhand großer, multizentrischer und internationaler Kohorten Evidenz für eine Therapieentscheidung bei bestimmten Patientensubgruppen zu schaffen. Der beeindruckende Datensatz mit einer langen Beobachtungszeit wurde statistisch detailliert und hochqualitativ analysiert. Diese Analysen zeigen beeindruckende Ergebnisse mit einer möglichen Reduktion des ACM-Risikos von bis zu 17 % für Patienten, die einen Tumor ≥ pT3a und einen Gleason-Score von 8 bis 10 nach RP aufwiesen. Wie die Autoren in der Diskussion korrekterweise feststellen, könnte eine undifferenzierte Interpretation der Evidenz der RCT zu der Schlussfolgerung verleiten, dass keinem Patienten eine aRT angeboten werden sollte. Auf Basis der beschriebenen Ergebnisse scheint uns eine solche Empfehlung nicht sinnvoll. Unsere Empfehlung ist, bestimmten Patientensubgruppen eine aRT nach RP anzubieten, da diese möglicherweise mit einer Reduktion des ACM-Risikos verbunden ist.

Dennoch ist eine solche Therapieentscheidung unter Berücksichtigung der individuellen Situation des Patienten sorgfältig abzuwägen. Die hier kommentierte Arbeit liefert leider keine Angaben zu den Toxizitäten und zur Lebensqualität nach den verschiedenen Therapien. Die RCT zeigten nämlich vermehrt Toxizitäten bei Durchführung einer aRT. Allerdings müssen bei der Beurteilung der Nebenwirkungen auch die teils veralteten RT-Techniken der RCT berücksichtigt werden. Zudem steht heutzutage mit der PSMA-PET/CT eine neue Methode zur Verfügung, die eine bessere Diagnostik in der Rezidiv- und Primärsituation ermöglicht. Ein Teil der in der kommentierten Studie inkludierten Patienten wäre möglicherweise heute nach Anwendung der üblichen Methoden bereits initial als Träger eines metastasierten PCa eingestuft und ganz anders behandelt worden.

Die PSMA-PET/CT ermöglicht zudem eine verbesserte Rezidivlokalisierung und damit im Falle einer sRT eine gezielte metastasengerichtete Behandlung und Anpassung der Bestrahlungsfelder je nach Metastasierungsmuster. Die möglichen Vorteile einer solchen Behandlung kann nach aktueller Evidenzlage nicht abschließend beantwortet werden, wenngleich es vielversprechende Ergebnisse einer metastasengerichteten Strahlentherapie gibt [5, 6]. Zukünftige Studien, welche die heutigen diagnostischen Möglichkeiten implementieren, könnten die Frage nach der optimalen Therapie bzw. eines verbesserten und individualisierten Strahlenfelds ebenso beantworten wie die nach der Anwendung und Dauer einer ADT. In diesem Zusammenhang sind Modulationen der Immunantwort durch RT und ADT und deren potenzieller klinischer Nutzen von besonderem Interesse und in Zukunft weiter zu erforschen. Zudem sind genomische Klassifizierungen weitere Werkzeuge, welche zur Risikoabschätzung und optimalen postoperativen Therapieentscheidung beitragen können, wenngleich ihre Rolle in diesem Zusammenhang, wie von den Autoren erwähnt, noch zu klären ist.

Zuletzt lässt sich eine weitere, provozierende Frage aus den präsentierten Daten ableiten. Unter der Annahme, dass bestimmte Patientensubgruppen mit lokalisiertem PCa eine adjuvante RT +/− Hormontherapie erhalten sollten, sollte geklärt werden, ob diese Patienten nicht initial mit einer primären Strahlentherapie besser und sicherer behandelt worden wären. Viele randomisierte Phase-III-Studien konnten bekanntlich exzellente Ergebnisse beim biochemischen rezidivfreien Überleben nach primärer perkutaner Strahlentherapie vorweisen. Eine umfassende, retrospektive Analyse zeigte zudem, dass die Kombination von Brachytherapie und perkutaner Bestrahlung bessere onkologische Ergebnisse verspricht als ein multimodaler Therapieansatz inklusive RP und darüber hinaus zu einer Reduktion distanter Metastasen führt [7]. Darüber hinaus kann der Antitumoreffekt der perkutanen Bestrahlung noch durch eine fokale Dosiseskalation der dominanten intraprostatischen Läsionen erhöht werden, ohne Nebenwirkungen zu verstärken, und zeigt auch bei Hochrisiko-PCa-Patienten rezidivfreie Überlebensraten von > 90 % [8]. Dieser Therapieansatz wird möglicherweise durch die Implementierung der PSMA-PET aufgrund einer verbesserten Tumorabdeckung noch weiter optimiert werden, sodass auch PCa-Patienten mit hohem Risiko, für die eine Brachytherapie nicht infrage kommt, mithilfe der RT effektiv behandelt werden können.

## Fazit

- Die Ergebnisse der hier besprochenen großen internationalen Studie belegen, dass Patienten mit einem ≥ pT3a-Tumor und Gleason-Score von 8 bis 10 mit oder ohne pN1 nach radikaler Prostatektomie eine Reduktion der „all-cause mortality“ nach adjuvanter RT im Vergleich zur Early-salvage-RT aufweisen. Diese bietet, entgegen den Ergebnissen der drei RCT (kontrollierte, randomisierte Phase-III-Studien) (GETUG-AFU 17, RADICALS-RT und RAVES) klare Evidenz, dass diesen Patientensubgruppen eine adjuvante RT angeboten werden sollte.
- Bei der Therapieentscheidung sind mögliche Toxizitäten und Fortschritte der bildgebenden Diagnostik zu berücksichtigen.
- In der PSMA-PET-Ära lassen sich die Art der postoperativen Therapie, die Ausdehnung des Bestrahlungsvolumens und die ADT weitergehend optimieren.

*Simon K.B. Spohn und Anca-Ligia Grosu, Freiburg, D*
